# Optimal Hematoma Volume Cut Points to Predict Functional Outcome After Basal Ganglia and Thalamic Hemorrhages

**DOI:** 10.3389/fneur.2018.00291

**Published:** 2018-05-01

**Authors:** Kazuma Nakagawa, Sage L. King, Todd B. Seto

**Affiliations:** ^1^The Queen’s Medical Center, Honolulu, HI, United States; ^2^Department of Medicine, John A. Burns School of Medicine, University of Hawaii, Honolulu, HI, United States

**Keywords:** intracerebral hemorrhage, cohort study, prognosis, basal ganglia, thalamus

## Abstract

**Background:**

Basal ganglia hemorrhage (BG-ICH) and thalamic hemorrhage (TH-ICH) have been historically grouped into a single “deep” hemorrhage group in prior studies. We aimed to assess whether BG-ICH and TH-ICH have different optimal hematoma volume cut points in predicting functional outcome.

**Methods:**

Patients with BG-ICH and TH-ICH with no preexisting disabilities who were enrolled in a single-center intracerebral hemorrhage (ICH) cohort study were studied. The hematoma volume of patients who achieved modified Rankin Scale (mRS) of ≤2 and ≤3 at 3 months were compared between BG-ICH and TH-ICH groups. Receiver operating characteristic (ROC) curves were created to determine the optimal hematoma volume cut points in predicting 3-month mRS of ≤2 and ≤3 for BG-ICH and TH-ICH groups.

**Results:**

A total of 135 (81 BG-ICH and 54 TH-ICH) patients were studied. The hematoma volume among those with 3-month mRS ≤ 2 (BG-ICH: 9.5 ± 5.4 cm^3^ vs. TH-ICH: 5.1 ± 4.9 cm^3^, *p* = 0.01) and 3-month mRS ≤ 3 (BG-ICH: 14.2 ± 13.4 cm^3^ vs. TH-ICH: 4.7 ± 4.1 cm^3^, *p* = 0.001) were smaller in TH-ICH than BG-ICH. The area under the ROC curve in predicting mRS ≤ 2 was 0.838 for BG-ICH (optimal hematoma volume cut point: 18.0 cm^3^, sensitivity 72.1%, specificity 95.0%) and 0.802 for TH-ICH (optimal hematoma volume cut point: 4.6 cm^3^, sensitivity 83.8%, specificity 70.6%); and in predicting mRS ≤ 3 was 0.826 for BG-ICH (optimal hematoma volume cut point: 28.8 cm^3^, sensitivity 71.4%, specificity 93.8%) and 0.902 for TH-ICH (optimal hematoma volume cut point: 5.5 cm^3^, sensitivity 92.9%, specificity 76.9%).

**Conclusion:**

TH-ICH have smaller optimal hematoma volume cut points than BG-ICH in predicting functional outcome.

## Introduction

Intracerebral hemorrhage (ICH) is a hemorrhagic stroke that results in disproportionately high mortality and morbidity (1). Favorable functional outcome at 3 months is seen only in 21–31% of the ICH population (2–4). Historically, basal ganglia hemorrhage (BG-ICH) and thalamic hemorrhage (TH-ICH) have been aggregated into a “deep” or “non-lobar” hemorrhage category in prior outcome prediction models (2, 4, 5).

Although basal ganglia and thalamus share a similar “deep” geographical location, their neuroanatomical functions differ substantially. Basal ganglia consist of caudate nucleus, putamen, globus pallidus, subthalamic nucleus, and substantia nigra and serve as a major center in the complex extrapyramidal system. The connections between basal ganglia and cortex help regulate automatic and voluntary responses and play a role in the emotional, motivational, associative, and cognitive motor planning (6). Compared to basal ganglia, the thalamus is smaller in size (approximately 3.0 cm × 2.0 cm × 2.0 cm), yet densely packed with 50–60 nuclei (6). Thalamus is conceptually regarded as a relay center for motor and sensory mechanisms, awareness, attention, and other neurocognitive processes including language and memory (6–8). Since thalamus has more neurocognitive functions and more nuclei per volume than basal ganglia, we hypothesized that hematoma volume cut point to predict favorable functional outcome is smaller in TH-ICH than BG-ICH.

## Materials and Methods

This study was approved by the University of Hawaii Institutional Review Board and written informed consent was obtained from the patient or legally authorized surrogate decision-maker. A single-center, multiethnic prospective cohort study of ICH patients (Queen Emma Stroke Study) was conducted from July 2011 to June 2016 at The Queen’s Medical Center (QMC) with a primary aim to assess ethnic disparities in long-term functional outcome after ICH. The inclusion and exclusion criteria and study protocol have been previously published (9). Management of ICH at QMC was in accordance with the most current guidelines (10, 11) at the time of patient enrollment. Last patient enrollment occurred in June 2015.

For this study, only those with BG-ICH and TH-ICH who were enrolled in the cohort study were included in the analyses. Patients lost to follow-up with no 3-month outcome data were excluded. Those with preexisting disabilities [modified Rankin Scale (mRS) > 0] were also excluded. Race was categorized as Whites, Asians, Native Hawaiians and other Pacific Islanders, or other. Clinical and radiographic information were abstracted from the electronic medical records and were confirmed by the patients, their family, or the treating medical team if needed. Initial systolic blood pressure, diastolic blood pressure, and Glasgow Coma Scale (GCS) score were obtained from the medical record. Do-not-resuscitate (DNR) orders were defined as any plan to limit cardiopulmonary resuscitation or mechanical ventilation in the event of a cardiopulmonary arrest. Based on the date/time of the DNR order entry, DNR status was categorized to “early DNR” (within 24 h of presentation) or “any DNR” (DNR orders at any time during the hospitalization). All head computed tomography (CT) scans were initially reviewed by one of the physician investigators, and hematoma volume was measured using the previously described ABC/2 method (12). Presence of intraventricular hemorrhage (IVH) and ICH neuroanatomical location were individually determined. To determine the location of ICH, posterior limb of the internal capsule was used as the anatomical border to differentiate basal ganglia from thalamus in the axial view of the head CT (anterior = basal ganglia; posterior = thalamus). When the ICH involved more than one anatomical location, an estimated location of the “epicenter” of the hematoma was used to classify the ICH location. The “epicenter” was defined as the point where the longest diameter (“A”) intersected the perpendicular longest diameter (“B”) in the CT slice with the largest area of hemorrhage (ABC/2 method). Furthermore, when more than half of the hemorrhage was seen posterior to the posterior limb of the internal capsule, it was considered TH-ICH; and *vice versa* for the BG-ICH. Outcome at 3 months was assessed by telephone or in-person interview using a standardized, simplified mRS questionnaire algorithm (13).

### Statistical Analysis

The clinical and radiographic characteristics of the BG-ICH group were compared with the TH-ICH group using the chi-square test for categorical data, two-tailed *t*-test for normally distributed, continuous variables, and Mann–Whitney *U* test for nonparametric data. Receiver operating characteristic (ROC) curves were created to determine the optimal hematoma volume cut points, sensitivity and specificity in predicting 3-month mRS of ≤2 and ≤3 for BG-ICH and TH-ICH groups. Data were analyzed using SPSS version 24.0 (SPSS IBM Inc., Chicago, IL, USA).

## Results

A total of 166 patients with deep hemorrhages were enrolled in the cohort study between July 2011 and June 2015. Among them, 15 patients did not have 3-month outcome data and 16 patients had baseline mRS > 0, and were excluded from the study. For the final analyses, 135 patients with deep hemorrhages (81 BG-ICH and 54 TH-ICH) with no preexisting disabilities were studied. Overall, 37 patients (27.4%) achieved mRS ≤ 2, 58 patients (43.0%) achieved mRS ≤ 3, and 46 patients (34.1%) patients were dead at 3 months. Table 1 provides the clinical comparison of BG-ICH and TH-ICH groups. TH-ICH patients were older, had higher prevalence of hypercholesterolemia, secondary IVH, and smaller hematoma volume. Median initial GCS was higher in TH-ICH than BG-ICH.

**Table 1:** Clinical characteristics of basal ganglia and TH-ICH patients.

	BG-ICH	TH-ICH	*p* Value
No. of patients	81	54	
Age, years	55.1 ± 16.9	64.1 ± 14.1	0.001
Female	33 (40.7)	17 (31.5)	0.28
Race			0.28
Whites	11 (13.6)	3 (5.6)	
Asians	48 (59.3)	39 (72.2)	
NHOPI	19 (23.5)	9 (16.7)	
Others	3 (3.7)	3 (5.6)	
Married	43 (53.1)	32 (59.3)	0.75
No health insurance	18 (22.2)	6 (11.3)	0.11
Annual household income <$15,000	27 (33.3)	14 (25.9)	0.36
Regular visit to a primary care physician	43 (55.1)	35 (70.0)	0.09
Hypertension (known history)	63 (77.8)	49 (90.7)	0.05
Diabetes mellitus	19 (23.5)	15 (27.8)	0.57
Hypercholesterolemia	25 (30.9)	27 (50.0)	0.03
Coronary artery disease	4 (4.9)	5 (9.3)	0.32
Atrial fibrillation	9 (11.1)	7 (13.0)	0.74
Prior stroke	5 (6.2)	8 (14.8)	0.10
Obesity (BMI ≥ 30 kg/m^2^)	35 (44.9)	15 (28.3)	0.06
Coagulopathy (INR ≥ 1.5)	8 (9.9)	6 (11.1)	0.82
Dementia	3 (3.7)	2 (3.7)	1.00
Methamphetamine abuse	18 (22.2)	5 (9.3)	0.05
Initial SBP, mmHg	183.9 ± 38.0	180.4 ± 33.8	0.59
Initial DBP, mmHg	105.4 ± 27.0	101.6 ± 22.5	0.40
Initial GCS, median [IQR]	12 [7, 15]	15 [7, 15]	0.02
DNR order, early (<24 h)	15 (18.5)	6 (11.1)	0.25
DNR order, any time during hospitalization	29 (35.8)	19 (35.2)	0.94
IVH	30 (37.0)	35 (64.8)	0.002
Hematoma volume (cm^3^)	44.4 ± 49.1	13.8 ± 16.9	<0.0001

*BG-ICH, basal ganglia hemorrhage; TH-ICH, thalamic hemorrhage; NHOPI, Native Hawaiians and other Pacific Islanders; BMI, body mass index; INR, international normalized ratio; SBP, systolic blood pressure; DBP, diastolic blood pressure; GCS, Glasgow Coma Scale; DNR, do-not-resuscitate; IVH, intraventricular hemorrhage*.

*Data are *n* (%), mean ± SD, or median [IQR]*.

The distribution of 3-month mRS were not significantly different between BG-ICH (mRS: 0: 3.7%; 1: 16.0%; 2: 4.9%; 3: 14.8%; 4: 18.5%; 5: 4.9%; and 6: 37.0%) and TH-ICH groups (mRS: 0: 1.9%; 1: 16.7%; 2: 13.0%; 3: 16.7%; 4: 11.1%; 5: 11.1%; and 6: 29.6%) (*p* = 0.39). The mean hematoma volume were significantly smaller in TH-ICH than BG-ICH among those who achieved mRS ≤ 2 at 3 months (mRS ≤ 2: BG-ICH: 9.5 ± 5.4 cm^3^ vs. TH-ICH: 5.1 ± 4.9 cm^3^, *p* = 0.01) and those who achieved mRS ≤ 3 at 3 months (mRS ≤ 3: BG-ICH: 14.2 ± 13.4 cm^3^ vs. TH-ICH: 4.7 ± 4.1 cm^3^, *p* = 0.001). Although the mean hematoma volume increased in the BG-ICH group when the definition of a favorable outcome was changed from mRS ≤ 2 to mRS ≤ 3, a similar trend was not observed in the TH-ICH group. When BG-ICH and TH-ICH were grouped together, the area under the ROC curve was 0.797 [95% confidence interval (CI): 0.724, 0.869] in predicting 3-month mRS ≤ 2 and 0.823 (95% CI: 0.754, 0.892) in predicting 3-month mRS ≤ 3. However, when BG-ICH and TH-ICH were analyzed individually, the area under the ROC curve improved (Table 2) suggesting that the predictive power of hematoma size enhances when the two groups are assessed separately. Based on the ROC curve, the optimal hematoma volume cut points to achieve the maximal sensitivity and specificity were 18.0 cm^3^ for BG-ICH and 4.6 cm^3^ for TH-ICH in predicting mRS ≤ 2; and 28.8 cm^3^ for BG-ICH and 5.5 cm^3^ for TH-ICH in predicting mRS ≤ 3 at 3 months.

**Table 2:** Association between hematoma volume and 3-month functional outcome.

	Area under the ROC curve (95% CI)	Sensitivity (%)	Specificity (%)	Optimal hematoma volume cut point (cm^3^)
**BG-ICH**				
mRS ≤ 2	0.838 (0.752, 0.924)	72.1	95.0	18.0
mRS ≤ 3	0.826 (0.733, 0.918)	71.4	93.8	28.8
**TH-ICH**				
mRS ≤ 2	0.802 (0.671, 0.933)	83.8	70.6	4.6
mRS ≤ 3	0.902 (0.824, 0.981)	92.9	76.9	5.5

*ROC, receiver operative characteristic; CI, confidence interval; BG-ICH, basal ganglia hemorrhage; TH-ICH, thalamic hemorrhage; mRS, modified Rankin Scale*.

## Discussion

Our study confirmed our hypothesis that TH-ICH has smaller hematoma volume cut points than BG-ICH in predicting favorable functional outcome. We used both mRS ≤ 2 and mRS ≤ 3 to define favorable functional outcome since both definitions have been used in ICH studies and may also have different clinical significance. The hematoma volume cut point of 30 cm^3^, which was initially derived by Broderick et al. (14) to predict 30-day mortality after ICH and later validated by the ICH Score (3, 15, 16) and FUNC Score (2), has become the most widely used hematoma volume cut point to predict outcome in the clinical settings, regardless of the neuroanatomical location. However, severely disabling outcome with a hematoma volume of <30 cm^3^ can be seen, especially after TH-ICH. A relatively small hemorrhage in thalamus could involve highly dense thalamic nuclei, and affect motor, sensory, and neurocognitive functions.

The intent of this study was to provide preliminary data suggesting the need to derive and validate more location-specific outcome prediction models to enhance the clinicians’ ability to prognosticate functional outcome after ICH. Prior ICH prognostication, models were initially derived to predict mortality (3, 14, 15), which was later applied to predict functional outcome. The FUNC Score, which was created to predict functional outcome, also used the hematoma volume cut point of <30, 30–60, and >60 cm^3^, after basing the cut points from the Broderick’s model, which was intended to predict mortality (14). The shortcomings of the two most widely used formal prediction scores, ICH Score and FUNC Score, were recently shown by Hwang et al. where the subjective clinical judgment of the physicians with neurological expertise were demonstrated to be superior than the two formal scores in predicting 3-month mRS (17). It is possible that the physicians in that study incorporated some knowledge of the fundamental neuroanatomical differences between basal ganglia and thalamus when predicting outcome.

In addition to providing short-term prognosis for the patients and their families, the neurologists are often asked to determine the likelihood of long-term disability after ICH. Perhaps, these new hematoma volume cut points shown in our study may help physicians prognosticate the patients’ chance of functional independence with higher certainty. This could be particularly useful in an environment with a shortage of physicians with neurological expertise since the primary care physicians or internists are frequently tasked to make these determinations.

The strength of our study is that this is the first study to specifically assess the relationship between hematoma volume and functional outcome separately among the BG-ICH and TH-ICH groups, making it more of a “location-specific” prognostication approach, rather than creating a “one size fits all” approach for all ICH. The limitation of our study is the small sample size, and single-centered nature of the study, which may limit the generalizability of our results to other populations. Due to the small sample size that limited the number of confounding variables that could be incorporated in the multivariable model, we were unable to derive a new outcome prediction model or enhanced ICH Score for BG-ICH and TH-ICH. We did not assess the severity of IVH, the need for ventriculostomy or ventriculoperitoneal shunt, and their associated surgical complications, which may have impacted the outcome. This was an exploratory study, and larger multi-center study is needed to derive and validate the enhanced outcome prediction models in BG-ICH and TH-ICH to confirm our findings.

In conclusion, this study showed that TH-ICH has smaller hematoma volume cut points than BG-ICH in predicting functional outcome. The functional outcome after ICH may variably manifest depending on the neuroanatomical location and highlights the importance of refining the previously established outcome prediction models to provide more specific and applicable tools that could be used in clinical settings.

## Ethics Statement

This study was approved by the University of Hawaii Institutional Review Board and written informed consent was obtained from the patient or legally authorized surrogate decision-maker.

## Author Contributions

KN participated in the conception and design of the study, the acquisition of data, the analysis and interpretation of data, and was responsible for drafting and finalizing the manuscript. SK participated in the acquisition of data and helped to draft and finalize the manuscript. TS participated in the study supervision, analysis and interpretation of data, and was responsible for drafting and finalizing the manuscript.

## Conflict of Interest Statement

The authors declare that the research was conducted in the absence of any commercial or financial relationships that could be construed as a potential conflict of interest.
